# Temporal changes in etiology, complications, and infection-related admissions among patients with cirrhosis: a 20-year single-center study

**DOI:** 10.3389/fmed.2026.1785288

**Published:** 2026-05-20

**Authors:** Rong Su, Wentun Yao, Yuchen Mian, Yanting Zhang, Shaoqi Yang

**Affiliations:** Department of Gastroenterology, General Hospital of Ningxia Medical University, Yinchuan, China

**Keywords:** cirrhosis, decompensated cirrhosis, etiological spectrum, infection-related admissions, real-world study, spontaneous bacterial peritonitis, temporal trends

## Abstract

**Background:**

The epidemiology and clinical presentation of cirrhosis have changed substantially over recent decades, driven by advances in antiviral therapy and shifts in the burden of chronic liver diseases. However, long-term real-world data describing concurrent changes in etiology, complications, and infection-related hospitalizations among patients with cirrhosis remain limited. This study was designed based on this background and these unmet clinical needs. By conducting a retrospective analysis of patients with liver cirrhosis hospitalized for the first time over more than 20 years at a tertiary medical center, this study aims to fill the gap in long-term real-world data in this field.

**Methods:**

In this retrospective observational study, adult patients hospitalized for cirrhosis for the first time between 2003 and 2025 at a tertiary medical center were included. Patients were grouped into five predefined periods according to the year of first hospitalization. Cirrhosis etiology, disease stage, and complications were systematically classified. Temporal changes in etiological composition and infection-related admissions were assessed descriptively, and trend analyses were performed where appropriate.

**Results:**

A total of 11,016 patients were analyzed. Viral-related cirrhosis remained the predominant etiology throughout the study period but declined significantly over time (93.12%–74.09%, *P* for trend < 0.001). In contrast, autoimmune- and metabolic/steatotic liver disease-related cirrhosis increased steadily (*P* for trend < 0.001 for both). Although most patients presented with decompensated cirrhosis at first hospitalization, the proportion of decompensated cases decreased over time. The spectrum of complications evolved across periods, with heterogeneous changes in decompensating events and portal hypertension–related manifestations. Infection-related admissions exhibited a structural shift: spontaneous bacterial peritonitis (SBP)–related admissions declined after the early 2010s, whereas non-SBP infection–related admissions increased gradually in recent years. In parallel, the proportion of patients diagnosed with hepatocellular carcinoma at first hospitalization decreased markedly over time.

**Conclusion:**

Over the past two decades, the etiological and clinical landscape of cirrhosis has evolved substantially. The declining dominance of viral-related cirrhosis, rising contributions of autoimmune and metabolic etiologies, and a shifting infection spectrum underscore the need for adaptive, etiology- and infection-oriented management strategies in contemporary cirrhosis care.

## 1 Introduction

Cirrhosis represents the final common pathway of chronic liver diseases and remains a major cause of morbidity and mortality worldwide (1). Over the past two decades, sustained progress in antiviral therapy, diagnostic techniques for autoimmune liver diseases, and management strategies for metabolic disorders has profoundly altered the disease spectrum and natural history of chronic liver diseases, thereby driving the dynamic evolution of the etiological composition and clinical manifestations of liver cirrhosis (2, 3).

The widespread application of direct-acting antivirals (DAAs) and nucleos(t)ide analogues has enabled the cure of hepatitis C and effective suppression of hepatitis B (4); the rising prevalence of obesity and diabetes has fueled the rapid spread of metabolic-associated fatty liver disease (MASLD) (5); and advancements in autoantibody detection and imaging technologies have improved the diagnostic rate of autoimmune liver diseases (6). Collectively, these developments have reshaped the etiological composition and clinical characteristics of patients with liver cirrhosis (2).

These shifts in etiology may have important implications for disease progression, complication patterns, and infection risk among patients with cirrhosis (7). The declining proportion of viral hepatitis-related cirrhosis theoretically reduces the long-term cumulative risk of hepatocellular carcinoma (HCC), as sustained viral suppression can interrupt the progression chain of inflammation-fibrosis-carcinogenesis (8). Patients with liver cirrhosis have a high incidence of bacterial infections, accounting for 25%–46% of hospitalization causes, with spontaneous bacterial peritonitis (SBP) being the predominant type (25%–31%) (9). Meanwhile, MASLD-related cirrhosis not only progresses to end-stage liver disease but also leads to significant extrahepatic complications such as cardiovascular disease, chronic kidney disease, and sarcopenia, with a risk profile significantly different from that of viral cirrhosis (10).

Infections are a leading cause of hospitalization and death in patients with cirrhosis (11). While SBP has traditionally been the most prominent infection in decompensated cirrhosis (12). However, recent studies suggest that the infection spectrum is changing, with a gradual increase in the proportion of non-SBP infections (such as urinary tract infections, pneumonia, and bloodstream infections) (13, 14). From a clinical practice perspective, this change has a logical basis–the widespread adoption of antibiotic prophylaxis strategies has reduced the incidence of SBP (15); meanwhile, increased opportunities for invasive diagnostic and therapeutic procedures, prolonged hospital stays, and longer survival times for patients with more severe disease in patients with liver cirrhosis have collectively increased the risk of non-SBP infections (16).

Based on the aforementioned background, this study conducted a retrospective observational analysis of patients with liver cirrhosis hospitalized for the first time over more than 20 years at a tertiary medical center. The strength of this study lies in its long-term span (2003–2025), which, unlike most short-term studies covering 5–10 years, more comprehensively captures the long-term impact of the widespread adoption of antiviral therapy, the prevalence of metabolic diseases, and advancements in diagnostic and therapeutic techniques on the clinical characteristics of liver cirrhosis. It clearly reveals a structural shift in the etiological spectrum from “virus-dominated” to “coexistence of viral, autoimmune, and metabolic etiologies,” providing long-term evidence for understanding the epidemiological changes in liver cirrhosis. The study also focuses on the coordinated changes in the etiological spectrum, complications, and infection-related hospitalizations. This multidimensional analysis offers a new perspective for exploring the complex clinical trajectories of liver cirrhosis. By providing this long-term real-world evidence, this study aims to furnish a basis for etiology-oriented and infection-oriented stratification strategies in the management of liver cirrhosis.

## 2 Materials and methods

### 2.1 Study design and population

This was a retrospective observational study conducted at a tertiary medical center. Consecutive patients hospitalized with a diagnosis of cirrhosis between January 2001 and December 2025 were screened using electronic medical records.

This study was reviewed and approved by the Medical Research Ethics Committee of General Hospital of Ningxia Medical University (approval number: KYLL-2024-0593). Due to the retrospective nature of the study and the use of anonymized clinical data, the requirement for informed consent was waived.

Patients were eligible for inclusion if they met the following criteria:

① Age ≥ 18 years at the time of hospitalization;
② Diagnosis of cirrhosis based on clinical, laboratory, imaging, endoscopic, and/or histological findings;
③ First hospitalization for cirrhosis during the study period.

Patients were excluded if they:

① Were younger than 18 years;
② Had cirrhosis secondary to Wilson’s disease (hepatolenticular degeneration);
③ Had incomplete key clinical data required for etiological or complication classification, including missing core data for etiological diagnosis: such as viral hepatitis markers [hepatitis B surface antigen (HBsAg)/anti-hepatitis C virus (HCV)], autoimmune liver disease antibodies [antinuclear antibody (ANA)/antimitochondrial antibody (AMA)], metabolic syndrome-related indicators (blood glucose/blood lipids/imaging data, etc.); insufficient evidence for the diagnosis and classification of complications such as imaging evidence of ascites, clinical grading records of hepatic encephalopathy, endoscopic reports of esophageal and gastric varices, etc.

For patients with multiple hospitalizations, only the first hospitalization was included to avoid duplication. The final study population consisted of 11,016 patients.

### 2.2 Definition of study periods

Patients were categorized into five calendar periods according to the year of first hospitalization: 2003–2007, 2008–2012, 2013–2017, 2018–2022, and 2023–2025.

Although data collection started in 2001, the first 2 years (2001–2002) were not included in the 5-years period–based analyses because they did not constitute a complete time interval and were characterized by relatively small annual case numbers (2001: 104 cases, 2002: 131 cases). To avoid potential instability and temporal misclassification in period-based comparisons, these years were treated as a run-in phase and were included only in analyses of annual trends.

The study period covered the COVID-19 pandemic from 2020 to 2023. Due to the limitations of the retrospective research design, patients’ COVID-19 infection history and vaccination status were not routinely collected. For data after 2020, only some patients had clear COVID-19 infection records, and the data integrity was insufficient, so it was not included in the main analysis.

### 2.3 Etiological classification of cirrhosis

The etiology of cirrhosis was determined based on comprehensive clinical evaluation and categorized as follows (17):

Viral-related cirrhosis: chronic hepatitis B virus (HBV) and/or HCV infection. Diagnosis was based on a confirmed history of chronic viral infection, positive serological markers (HBsAg or anti-HCV) or viral nucleic acid tests (HBV DNA or HCV RNA), after the exclusion of other identifiable etiologies.

Autoimmune-related cirrhosis: autoimmune hepatitis (AIH), primary biliary cholangitis (PBC), primary sclerosing cholangitis, or AIH–PBC overlap syndrome. Diagnosis was confirmed by autoantibody testing (e.g., ANA, AMA, antineutrophil cytoplasmic antibody), characteristic liver function profiles, and histological examination.

Alcohol-related cirrhosis: history of significant alcohol consumption with exclusion of other dominant causes; specifically, a drinking history of ≥5 years, with an average daily alcohol intake of ≥40 g for males and ≥20 g for females.

Metabolic/steatotic-related cirrhosis: cirrhosis attributed to non-alcoholic fatty liver disease or metabolic dysfunction-associated fatty liver disease. Diagnosis was based on the presence of metabolic syndrome-related manifestations (obesity, diabetes, hyperlipidemia, etc.), evidence of steatohepatitis confirmed by metabolic indicators (e.g., blood lipids, blood glucose), imaging, or histology, after excluding alcohol and other definite etiologies.

Drug-induced cirrhosis: chronic liver injury attributed to medications or toxic exposures. Diagnosis was supported by a clear history of drug/toxic exposure, specific testing, or histological evidence.

Cryptogenic/unknown cirrhosis: cirrhosis with no identifiable cause after standard evaluation.

When multiple etiological factors were present, the primary etiology was determined according to the dominant clinical cause and the chronological sequence of disease onset: (1) The dominant clinical cause: Priority was given to factors that played a decisive role in the occurrence and development of liver cirrhosis. For example, for patients with chronic HBV/HCV infection even if combined with a drinking history, if the liver histological manifestations were mainly viral hepatitis damage, viral-related cirrhosis was still considered the primary diagnosis. (2) The chronological sequence of disease onset: Judgment was made based on the time sequence of etiology exposure and liver disease progression. For example, when a long-term drinking history precedes the appearance of metabolic abnormalities, alcohol-related etiology is given priority. (3) Exclusion method: When it is impossible to identify a single dominant factor, other more common or more pathogenic factors (such as viral infection and alcohol) are excluded before considering autoimmune, metabolic, or cryptogenic etiologies.

### 2.4 Definition of disease stage and decompensating events

Cirrhosis was classified as decompensated if patients had experienced at least one decompensating event prior to or at the time of first hospitalization.

Decompensating events were defined as the occurrence of any of the following: clinically significant ascites, esophagogastric variceal bleeding, hepatic encephalopathy, or SBP.

For each patient, recurrent episodes of the same event type were counted as a single decompensating event. The number of decompensating events represented the number of distinct event types experienced (0, 1, or ≥2) (17).

### 2.5 Definition of complications

Complications were classified into the following categories:

Decompensating events: ascites, variceal bleeding, hepatic encephalopathy, and SBP; Portal hypertension–related complications: esophagogastric varices, hypersplenism/thrombocytopenia, and portal vein thrombosis; Infection-related complications: infections requiring hospitalization excluding SBP (non-SBP infections), including respiratory tract infections (such as lung infections), urinary tract infections, skin and soft tissue infections (such as cellulitis), sepsis, other abdominal infections (such as biliary tract infections), etc.; Malignant outcome: HCC diagnosed at or before first hospitalization. Patients could have more than one complication (17, 18).

### 2.6 Statistical analysis

Categorical variables were expressed as numbers (percentages), with percentages calculated using the total number of patients in each period as the denominator. Continuous variables were summarized using median and interquartile range, where applicable.

Temporal trends in etiological proportions across periods were assessed using the Cochran–Armitage trend test. Annual trends were illustrated graphically.

All statistical analyses were performed using SPSS software (25.0, IBM Corp.), and a two-sided *P*-value < 0.05 was considered statistically significant.

## 3 Results

### 3.1 Study population and baseline characteristics

A total of 11,016 patients with cirrhosis who were hospitalized for the first time between 2003 and 2025 were included in the analysis. Patients were stratified into five periods: 2003–2007 (*n* = 1,425), 2008–2012 (*n* = 2,946), 2013–2017 (*n* = 3,063), 2018–2022 (*n* = 2,178), and 2023–2025 (*n* = 1,404).

The proportion of male patients showed a gradual decline over time, decreasing from 73.61% in 2003–2007 to 60.47% in 2023–2025. Viral-related cirrhosis remained the predominant etiology throughout the study period; however, its proportion decreased steadily from 93.12% in 2003–2007 to 74.09% in 2023–2025. In contrast, autoimmune-related cirrhosis increased continuously, rising from 3.72% to 17.08% across the same periods.

Alcohol-related and drug-induced cirrhosis accounted for a relatively small proportion of cases in all periods, with no clear monotonic trend. Metabolic/steatotic-related cirrhosis demonstrated a marked increase, particularly after 2013, rising from 0.21% in 2003–2007 to 4.27% in 2023–2025. The proportion of cryptogenic/unknown cirrhosis increased initially and subsequently declined in the most recent period.

At first hospitalization, the majority of patients presented with decompensated cirrhosis. Although nearly all patients were decompensated in earlier periods, the proportion of decompensated cirrhosis decreased from 100% in 2003–2007 to 87.32% in 2023–2025. Regarding the number of decompensating events, the proportion of patients with no prior decompensating events increased over time, whereas those experiencing one or multiple distinct decompensating events showed temporal variation across periods (Table 1).

**TABLE 1:** Baseline characteristics and complication spectrum of patients with cirrhosis at first hospitalization by period.

Baseline characteristics	2003–2007	2008–2012	2013–2017	2018–2022	2023–2025	*P* for trend
	1425	2946	3063	2178	1404	
Demographics						
Age, years, median (IQR)	55 (44.64)	51 (43.61)	52 (44.61)	55 (47.77)	57 (49.78)	<0.001
Male sex, *n* (%)	1049 (73.61)	2128 (72.23)	2131 (69.57)	1410 (64.74)	849 (60.47)	<0.001
Etiology						<0.001
Viral-related cirrhosis, *n* (%)	1327 (93.12)	2588 (87.85)	2567 (83.78)	1715 (78.74)	1041 (74.09)	<0.001
Autoimmune-related cirrhosis, *n* (%)	53 (3.72)	231 (7.84)	259 (8.45)	299 (13.73)	240 (17.08)	<0.001
Alcohol-related cirrhosis, *n* (%)	15 (1.05)	76 (2.58)	55 (1.80)	30 (1.38)	30 (2.14)	0.083
Metabolic/steatotic-related cirrhosis, *n* (%)	3 (0.21)	3 (0.10)	17 (0.55)	34 (1.56)	60 (4.27)	<0.001
Drug-induced cirrhosis, *n* (%)	5 (0.35)	7 (0.24)	8 (0.26)	4 (0.18)	3 (0.21)	0.742
Cryptogenic/unknown cirrhosis, *n* (%)	22 (1.54)	41 (1.39)	157 (5.12)	96 (4.41)	30 (2.14)	<0.001
Disease stage						
Decompensated cirrhosis, *n* (%)	1425 (100)	2930 (99.46)	2962 (96.70)	1949 (89.49)	1226 (87.32)	<0.001
Number of decompensating events, *n* (%)						<0.001
0	130 (9.12)	188 (6.38)	788 (25.73)	455 (20.89)	283 (20.16)	<0.001
1	863 (60.56)	1980 (67.21)	1512 (49.36)	984 (45.18)	728 (51.85)	<0.001
≥2	432 (30.32)	778 (26.41)	763 (24.91)	739 (33.93)	393 (27.99)	<0.001
Type of complications, n (%)						
Decompensating events						
Ascites	396 (27.8)	699 (23.7)	593 (19.4)	279 (12.8)	270 (19.2)	<0.001
Variceal bleeding	539 (37.8)	975 (33.1)	909 (29.7)	986 (45.3)	676 (48.2)	<0.001
Hepatic encephalopathy	301 (21.1)	543 (18.4)	425 (13.9)	359 (16.5)	251 (17.9)	<0.001
Spontaneous bacterial peritonitis (SBP)	159 (11.2)	558 (18.9)	352 (11.5)	130 (6.0)	110 (7.8)	<0.001
Portal hypertension–related						
Esophagogastric varices	67 (4.7)	105 (3.6)	219 (7.2)	182 (8.4)	157 (11.2)	<0.001
Hypersplenism/thrombocytopenia	38 (2.7)	69 (2.3)	247 (8.1)	138 (6.3)	14 (1.0)	<0.001
Portal vein thrombosis (optional)	25 (1.8)	14 (0.5)	9 (0.3)	10 (0.5)	4 (0.3)	<0.001
Infection-related						
Any infection (excluding SBP)	43 (3.0)	55 (1.9)	87 (2.8)	76 (3.5)	61 (4.3)	<0.001
Malignant outcome						
Hepatocellular carcinoma	281 (19.7)	582 (19.8)	235 (7.7)	59 (2.7)	51 (3.6)	<0.001

Values are presented as *n* (%), with percentages calculated using the total number of patients in each period as the denominator. *P* for trend was calculated using the Cochran–Armitage trend test (519.86). All patients were hospitalized at a tertiary center, and the majority presented with decompensated cirrhosis at first admission. Decompensating events included clinically significant ascites, esophagogastric variceal bleeding, hepatic encephalopathy, and spontaneous bacterial peritonitis. For each patient, recurrent episodes of the same type were counted as one event, and the number of decompensating events represents the number of distinct event types experienced. Patients could have more than one complication.

### 3.2 Temporal changes in etiological spectrum of cirrhosis

Significant temporal changes were observed in the etiological spectrum of cirrhosis across the five study periods (Table 1). The proportion of viral-related cirrhosis showed a significant downward trend over time (*P* for trend < 0.001). Conversely, autoimmune-related cirrhosis demonstrated a consistent and significant upward trend (*P* for trend < 0.001).

Metabolic/steatotic-related cirrhosis increased markedly over time, particularly in the most recent periods (*P* for trend < 0.001). Cryptogenic/unknown cirrhosis also exhibited a significant temporal changes (*P* for trend < 0.001). In contrast, no significant temporal trend was observed for alcohol-related cirrhosis (*P* for trend = 0.083) or drug-induced cirrhosis (*P* for trend = 0.742).

Subgroup analyses showed that among viral-related cirrhosis, HBV infection remained the dominant cause throughout all periods, whereas HCV-related cirrhosis increased modestly over time (Supplementary Table 1A). Within autoimmune-related cirrhosis, the proportion of AIH and overlap syndromes increased, while PBC accounted for a decreasing proportion in recent years (Supplementary Table 1B).

### 3.3 Temporal changes in complication spectrum

The spectrum of complications among patients with cirrhosis changed substantially over time (Table 1). Among decompensating events, the proportion of patients presenting with ascites declined progressively from 27.8% in 2003–2007 to 12.8% in 2018–2022, followed by a modest increase in 2023–2025 (19.2%). Variceal bleeding remained common throughout all periods and increased notably in the most recent periods.

The prevalence of hepatic encephalopathy decreased initially and then remained relatively stable, whereas SBP showed a peak during 2008–2012 followed by a decline in subsequent periods.

Portal hypertension–related complications demonstrated heterogeneous trends. The proportion of esophagogastric varices increased gradually over time, while hypersplenism/thrombocytopenia peaked during 2013–2017 and declined thereafter. Portal vein thrombosis remained uncommon across all periods.

Infection-related admissions excluding SBP accounted for a small but increasing proportion of hospitalizations in later periods. The proportion of patients diagnosed with HCC at first hospitalization declined markedly over time, decreasing from approximately 20% in earlier periods to less than 4% in 2023–2025.

### 3.4 Annual trends in etiological composition and infection-related admissions

**FIGURE 1:**
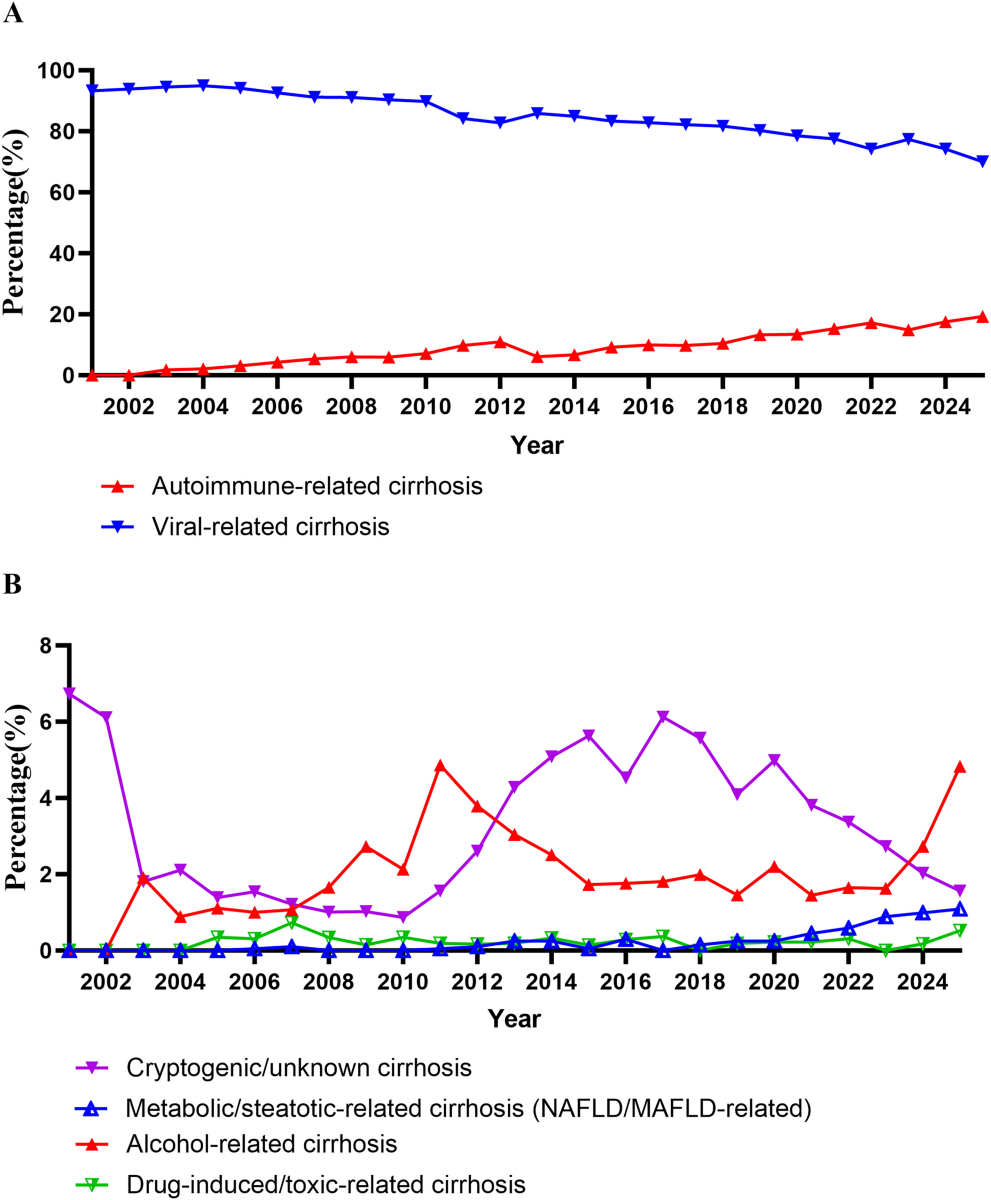
**(A)** Annual trends in the proportion of viral- and autoimmune-related cirrhosis among hospitalized patients with cirrhosis, 2001–2025. **(B)** Annual trends in non-viral etiological categories of cirrhosis, including metabolic/ steatotic-, alcohol-, drug-induced, and cryptogenic cirrhosis, 2001–2025.

Annual trend analyses further illustrated dynamic changes in cirrhosis etiology from 2001 to 2025 (Figure 1). Viral-related cirrhosis demonstrated a sustained decline, whereas autoimmune-related cirrhosis showed a continuous increase over the study period (Figure 1A). Metabolic/steatotic-related cirrhosis increased gradually, particularly after 2015, while alcohol-related, drug-induced, and cryptogenic cirrhosis exhibited relatively modest fluctuations (Figure 1B).

**FIGURE 2:**
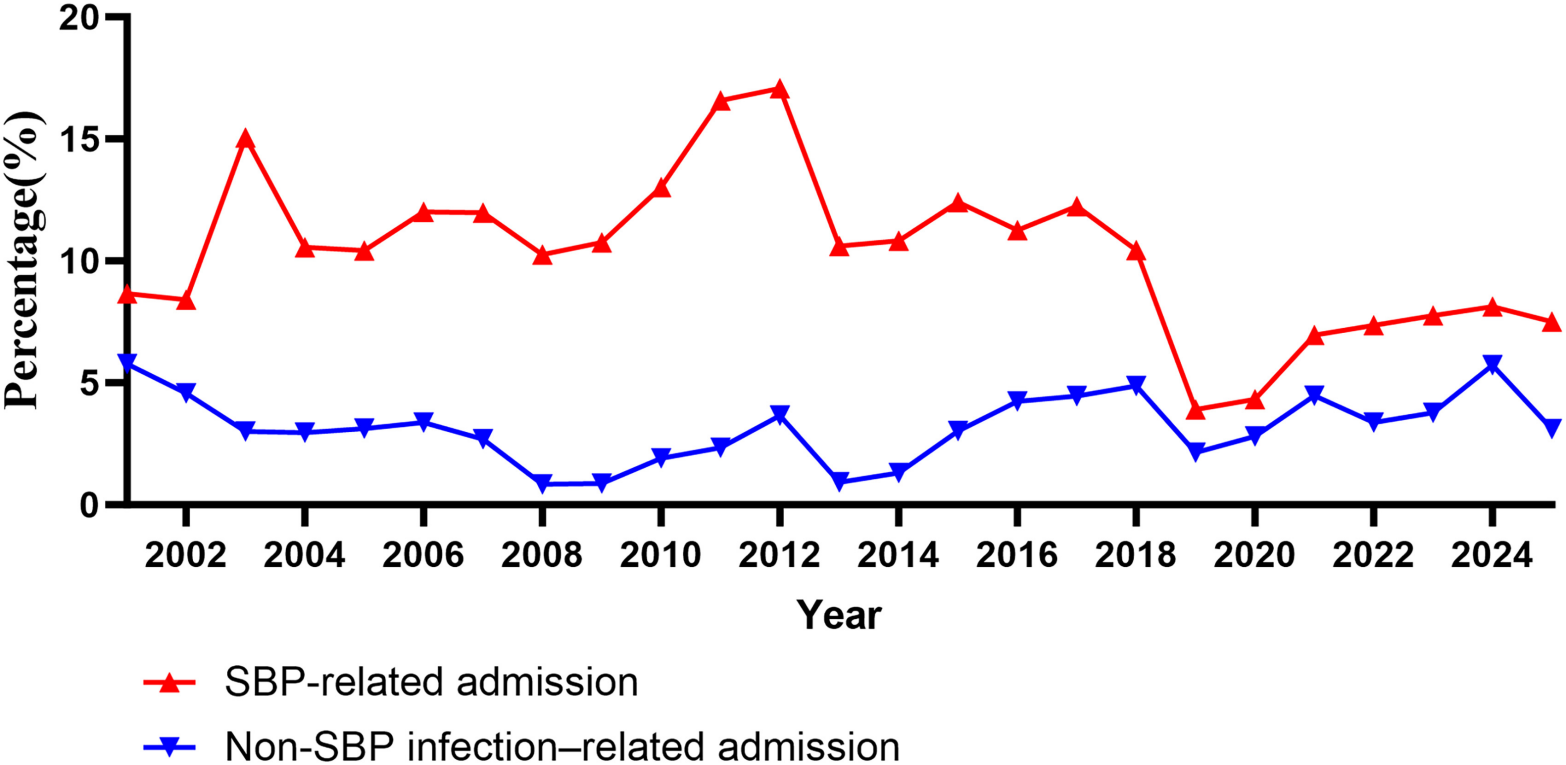
Annual trends in infection-related admissions among patients hospitalized for cirrhosis, stratified by spontaneous bacterial peritonitis (SBP) and non-SBP infections, 2001–2025.

Infection-related admissions also displayed distinct temporal patterns (Figure 2). SBP-related admissions accounted for a consistently higher proportion compared with non-SBP infections, with notable fluctuations over time. Non-SBP infection-related admissions showed a gradual upward trend in recent years.

## 4 Discussion

In this large, single-center retrospective study spanning more than two decades, we systematically characterized temporal changes in the etiological composition, complication spectrum, and infection-related admissions among patients hospitalized for cirrhosis. The results of this study indicated significant structural changes in the etiological spectrum, complication spectrum, and infection-related admission patterns among hospitalized patients with liver cirrhosis. The proportion of virus-related liver cirrhosis decreased from 93.12% to 74.09%, while autoimmune liver cirrhosis (3.72%→17.08%) and metabolic liver cirrhosis (0.21%→4.27%) showed a continuous upward trend. During the same period, the proportion of decompensation at first hospitalization decreased from 100% to 87.32%, the HCC diagnosis rate dropped from approximately 20% to less than 4%, and the infection spectrum also shifted from SBP to non-SBP infections. These descriptive results reflect the dynamic evolution of the disease spectrum of liver cirrhosis in the real world, providing important references for clinical practice and policy-making.

The continuous decline in the proportion of viral liver cirrhosis coincides temporally with significant advancements in antiviral therapy over the past two decades. The widespread clinical application of DAAs and nucleos(t)ide analogues has substantially alleviated the disease progression pressure of HBV- and HCV-related liver diseases (4). Meanwhile, the sustained increase in the global prevalence of obesity and diabetes has driven the rapid increase in MASLD-related liver cirrhosis (16). The rise in the proportion of autoimmune liver cirrhosis may reflect improvements in autoantibody detection technology and diagnostic awareness. Notably, this study found that the proportion of autoimmune liver cirrhosis increased more during the COVID-19 pandemic from 2020 to 2023. This observation is consistent with several recent studies (19, 20), suggesting a potential association between COVID-19 infection and an increased risk of autoimmune liver diseases. Potential mechanisms may include autoimmune reactions triggered by molecular mimicry between the spike protein of the coronavirus and liver tissue (21, 22); immune system hyperactivation and regulatory imbalance caused by COVID-19 infection (20, 21); and diagnostic delays due to reduced accessibility to medical resources during the pandemic. However, since this study did not systematically collect patients’ COVID-19 infection history and vaccination status, it cannot directly confirm this association, and the aforementioned mechanistic speculations require further prospective studies for validation.

Second, the significant decline in the HCC diagnosis rate at first hospitalization (approximately 20%→ < 4%) is an important manifestation of the long-term benefits of antiviral therapy. Sustained viral suppression can interrupt the progression chain of inflammation-fibrosis-carcinogenesis, thereby reducing the cumulative risk of HCC (23, 24). The decline in the proportion of decompensation may reflect improved early diagnostic capabilities or increased hospitalization opportunities for patients with milder disease. However, it is noteworthy that variceal bleeding has shown an upward trend recently–a phenomenon that may be related to the prolonged survival time of such patients or may reflect different bleeding risk profiles in patients with non-viral liver cirrhosis (such as MASLD-related liver cirrhosis) (25). The pattern of ascites proportion first decreasing and then increasing, and SBP peaking in the early 2010s and then declining, is consistent temporally with the promotion of standardized antibiotic prophylaxis strategies (26). It is also worth noting that the relative frequencies of specific decompensating events varied across periods, highlighting the heterogeneous clinical trajectories of cirrhosis in different eras.

Furthermore, this study found that although the proportion of hospitalizations related to non-SBP infections remains relatively low, it has shown a gradual upward trend in recent years, while SBP-related admissions have declined after peaking in the early 2010s. This structural shift in the infection spectrum may be driven by multiple factors: the widespread adoption of antibiotic prophylaxis strategies has effectively reduced the incidence of SBP, with a meta-analysis confirming that norfloxacin prophylaxis can reduce the risk of SBP (27). Meanwhile, increased opportunities for invasive diagnostic and therapeutic procedures and prolonged hospital stays in patients with liver cirrhosis are associated with an increased risk of infection (28). This study also observed a temporal coincidence between the rise in the proportion of autoimmune liver cirrhosis and the increase in non-SBP infections–patients with autoimmune liver diseases have a significantly increased risk of opportunistic infections and sepsis due to long-term use of immunosuppressants (29), which may shift the infection risk from typical SBP to other types, such as bacteremia and opportunistic infections.

Several strengths of this study merit consideration. The long observation period allowed for the assessment of secular trends across different therapeutic eras, and the use of first hospitalization minimized duplication and selection bias related to repeated admissions. In addition, etiological and complication classifications were based on comprehensive clinical evaluation, enabling a detailed characterization of disease patterns.

This study also has limitations. First, as a single-center retrospective observational study, the results have certain limitations in terms of extrapolation and generalizability; its main function is to reveal phenomena, describe associations, and explore trends, rather than directly establish causal relationships. Therefore, the temporal associations between changes in the etiological spectrum and external factors should be understood as speculative explanations based on temporal logic rather than causal inferences. Second, the completeness of infection-related data is insufficient. Retrospective medical records did not systematically record exposure time and location, and this study only included infection events at first hospitalization, excluding new-onset infections during hospitalization. Therefore, this study did not specifically subdivide non-SBP infections in the section “3 Results” or distinguish between community-acquired and hospital-acquired infections. Third, this study was designed as a cross-sectional characteristic analysis focusing on clinical characteristics at first hospitalization rather than long-term prognosis. Since it did not systematically collect post-discharge survival status, time of death, and cause of death, long-term survival analysis could not be conducted. Fourth, this study did not systematically collect patients’ COVID-19 infection history and vaccination status, making it impossible to directly assess the impact of the pandemic on patients with liver cirrhosis. Although a special increase in the proportion of autoimmune liver cirrhosis was observed during the pandemic, individual-level data were lacking to verify this association.

In conclusion, this study demonstrates substantial temporal changes in the etiology and clinical presentation of cirrhosis over the past two decades. The declining dominance of viral-related cirrhosis, coupled with the rising contribution of autoimmune and metabolic etiologies and a shifting infection spectrum, underscores the need for adaptive management strategies tailored to the evolving landscape of cirrhosis. The main strengths of this study lie in: ① adopting a long-term span of over 20 years (2003–2025) to capture the long-term impact of the widespread adoption of antiviral therapy, the prevalence of metabolic diseases, and advancements in diagnostic and therapeutic techniques on the characteristics of liver cirrhosis, revealing a structural shift in the etiological spectrum from “virus-dominated” to “the coexistence of viral, autoimmune, and metabolic etiologies”; ② focusing on the coordinated changes in the etiological spectrum, complications, and infection-related hospitalizations, finding that the rise in autoimmune liver cirrhosis may be associated with an increase in non-SBP infections, and the decline in viral liver cirrhosis is accompanied by a decrease in the HCC diagnosis rate, providing a new multidimensional perspective for understanding the clinical trajectories of liver cirrhosis. Future studies should be improved in the following aspects: First, adopt a multicenter prospective design, incorporating medical centers from different regions and types, to enhance the extrapolation of the results; second, systematically collect COVID-19 infection-related data (infection history, vaccination status, infection severity, etc.) to more comprehensively assess the impact of the pandemic on patients with liver cirrhosis; third, clearly distinguish between community-acquired infections, healthcare-associated infections, and hospital-acquired infections, and systematically record new-onset infection events during hospitalization; fourth, establish a standardized long-term follow-up system to collect patients’ survival status, time of death, and cause of death, and conduct prognostic analysis. Based on the findings of this study, the research team will design more targeted studies on specific etiologies (such as autoimmune liver cirrhosis, MASLD-related liver cirrhosis) or specific complications (such as non-SBP infections), and further explore causal relationships by collecting more detailed information on exposures and confounding factors.

## Data Availability

The raw data supporting the conclusions of this article will be made available by the authors, without undue reservation.
