# Supplementary material for: Temporal changes in etiology, complications, and infection-related admissions among patients with cirrhosis: a 20-year single-center study
Source: Front Med (Lausanne). 2026 May 20;13:1785288. doi: 10.3389/fmed.2026.1785288 (PMC13231285; doi:10.3389/fmed.2026.1785288)

**Table 1. Baseline characteristics and complication spectrum of patients with cirrhosis at first hospitalization by period**

|  | 2003–2007 | 2008–2012 | 2013–2017 | 2018–2022 | 2023–2025 | *P* for trend |
| --- | --- | --- | --- | --- | --- | --- |
|  | 1425 | 2946 | 3063 | 2178 | 1404 |  |
| Demographics |  |  |  |  |  |  |
| Age, years, median (IQR) | 55 (44,64) | 51 (43,61) | 52 (44,61) | 55 (47,77) | 57 (49,78) | <0.001 |
| Male sex, n (%) | 1049 (73.61) | 2128 (72.23) | 2131 (69.57) | 1410 (64.74) | 849 (60.47) | <0.001 |
| Etiology |  |  |  |  |  | <0.001 |
| Viral-related cirrhosis, n (%) | 1327 (93.12) | 2588 (87.85) | 2567 (83.78) | 1715 (78.74) | 1041 (74.09) | <0.001 |
| Autoimmune-related cirrhosis, n (%) | 53 (3.72) | 231 (7.84) | 259 (8.45) | 299 (13.73) | 240 (17.08) | <0.001 |
| Alcohol-related cirrhosis, n (%) | 15 (1.05) | 76 (2.58) | 55 (1.80) | 30 (1.38) | 30 (2.14) | 0.083 |
| Metabolic/steatotic-related cirrhosis, n (%) | 3 (0.21) | 3 (0.10) | 17 (0.55) | 34 (1.56) | 60 (4.27) | <0.001 |
| Drug-induced cirrhosis, n (%) | 5 (0.35) | 7 (0.24) | 8 (0.26) | 4 (0.18) | 3 (0.21) | 0.742 |
| Cryptogenic/unknown cirrhosis, n (%) | 22 (1.54) | 41 (1.39) | 157 (5.12) | 96 (4.41) | 30 (2.14) | <0.001 |
| Disease stage |  |  |  |  |  |  |
| Decompensated cirrhosis, n (%) | 1425(100) | 2930(99.46) | 2962(96.70) | 1949(89.49) | 1226(87.32) | <0.001 |
| Number of decompensating events, n (%) |  |  |  |  |  | <0.001 |
| 0 | 130 (9.12) | 188 (6.38) | 788 (25.73) | 455 (20.89) | 283 (20.16) | <0.001 |
| 1 | 863 (60.56) | 1980 (67.21) | 1512 (49.36) | 984 (45.18) | 728 (51.85) | <0.001 |
| ≥2 | 432 (30.32) | 778 (26.41) | 763 (24.91) | 739 (33.93) | 393 (27.99) | <0.001 |
| Type of complications，n (%) |  |  |  |  |  |  |
| **Decompensating events** |  |  |  |  |  |  |
| Ascites | 396 (27.8) | 699 (23.7) | 593 (19.4) | 279 (12.8) | 270 (19.2) | <0.001 |
| Variceal bleeding | 539 (37.8) | 975 (33.1) | 909 (29.7) | 986 (45.3) | 676 (48.2) | <0.001 |
| Hepatic encephalopathy | 301 (21.1) | 543 (18.4) | 425 (13.9) | 359 (16.5) | 251 (17.9) | <0.001 |
| Spontaneous bacterial peritonitis (SBP) | 159 (11.2) | 558 (18.9) | 352 (11.5) | 130 (6.0) | 110 (7.8) | <0.001 |
| **Portal hypertension–related** |  |  |  |  |  |  |
| Esophagogastric varices | 67 (4.7) | 105 (3.6) | 219 (7.2) | 182 (8.4) | 157 (11.2) | <0.001 |
| Hypersplenism / thrombocytopenia | 38 (2.7) | 69 (2.3) | 247 (8.1) | 138 (6.3) | 14 (1.0) | <0.001 |
| Portal vein thrombosis (optional) | 25 (1.8) | 14 (0.5) | 9 (0.3) | 10 (0.5) | 4 (0.3) | <0.001 |
| **Infection-related** |  |  |  |  |  |  |
| Any infection (excluding SBP) | 43 (3.0) | 55 (1.9) | 87 (2.8) | 76 (3.5) | 61 (4.3) | <0.001 |
| **Malignant outcome** |  |  |  |  |  |  |
| Hepatocellular carcinoma | 281 (19.7) | 582 (19.8) | 235 (7.7) | 59 (2.7) | 51 (3.6) | <0.001 |

Note: 1.Values are presented as n (%), with percentages calculated using the total number of patients in each period as the denominator. P for trend was calculated using the Cochran–Armitage trend test(519.86). 2.All patients were hospitalized at a tertiary center, and the majority presented with decompensated cirrhosis at first admission. 3.Decompensating events included clinically significant ascites, esophagogastric variceal bleeding, hepatic encephalopathy, and spontaneous bacterial peritonitis. For each patient, recurrent episodes of the same type were counted as one event, and the number of decompensating events represents the number of distinct event types experienced. 4. Patients could have more than one complication.

**Supplementary Table S1A. Viral-related cirrhosis subtypes**

|  | 2003–2007  (n=1327.) | 2008–2012  (n=2588) | 2013–2017  (n=2567) | 2018–2022  (n=1715) | 2023–2025  (n=1401) |
| --- | --- | --- | --- | --- | --- |
| HBV-related cirrhosis | 1324 (99.32) | 2571 (99.11) | 2501 (97.43) | 1682 (98.08) | 1004 (96.45) |
| HCV-related cirrhosis | 3 (0.68) | 11 (0.89) | 66 (2.57) | 33 (1.92) | 37 (3.55) |

**Supplementary Table S1B. Autoimmune-related cirrhosis subtypes**

|  | 2003–2007  (n=53) | 2008–2012  (n=231) | 2013–2017  (n=259) | 2018–2022  (n=299) | 2023–2025  (n=240) |
| --- | --- | --- | --- | --- | --- |
| AIH-related cirrhosis | 13 (24.5) | 36 (15.6) | 53 (20.5) | 103 (34.4) | 123 (51.2) |
| PBC-related cirrhosis | 38 (71.7) | 190 (82.3) | 186 (71.8) | 170 (56.9) | 90 (37.5) |
| PSC-related cirrhosis | 2 (3.8) | 5 (2.2) | 2 (0.8) | 4 (1.3) | 3 (1.3) |
| PBC+AIH | 0 (0.0) | 0 (0.0) | 18 (6.9) | 22 (7.4) | 24 (10.0) |


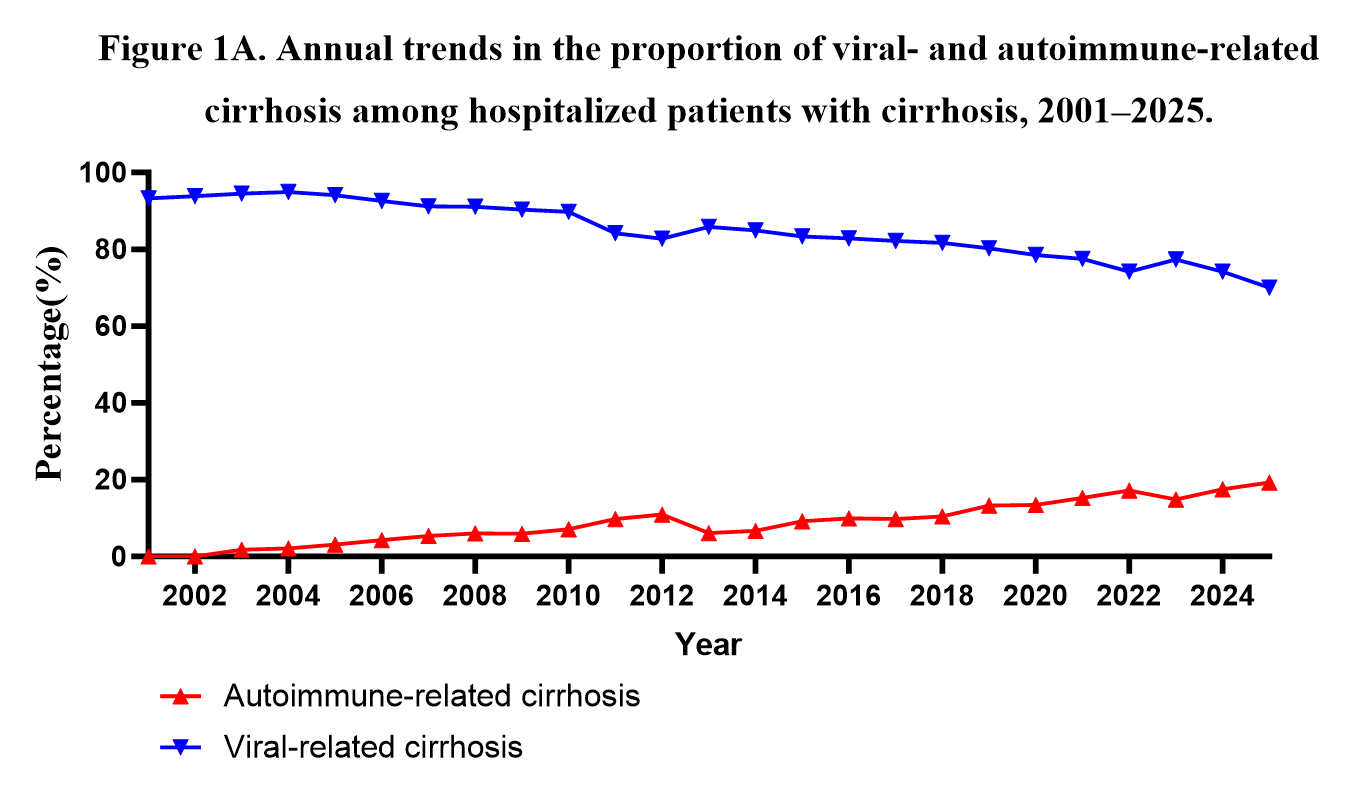


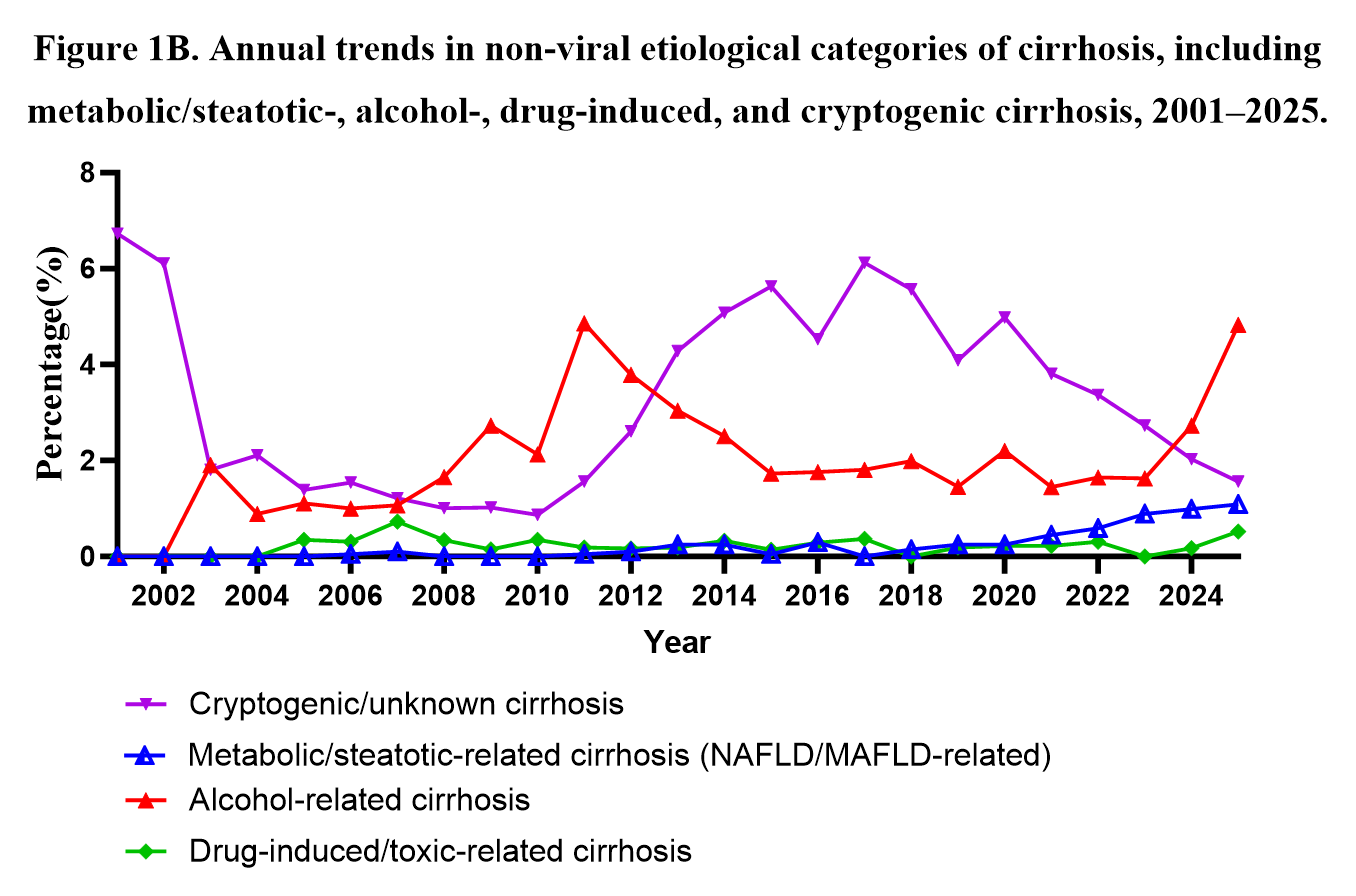


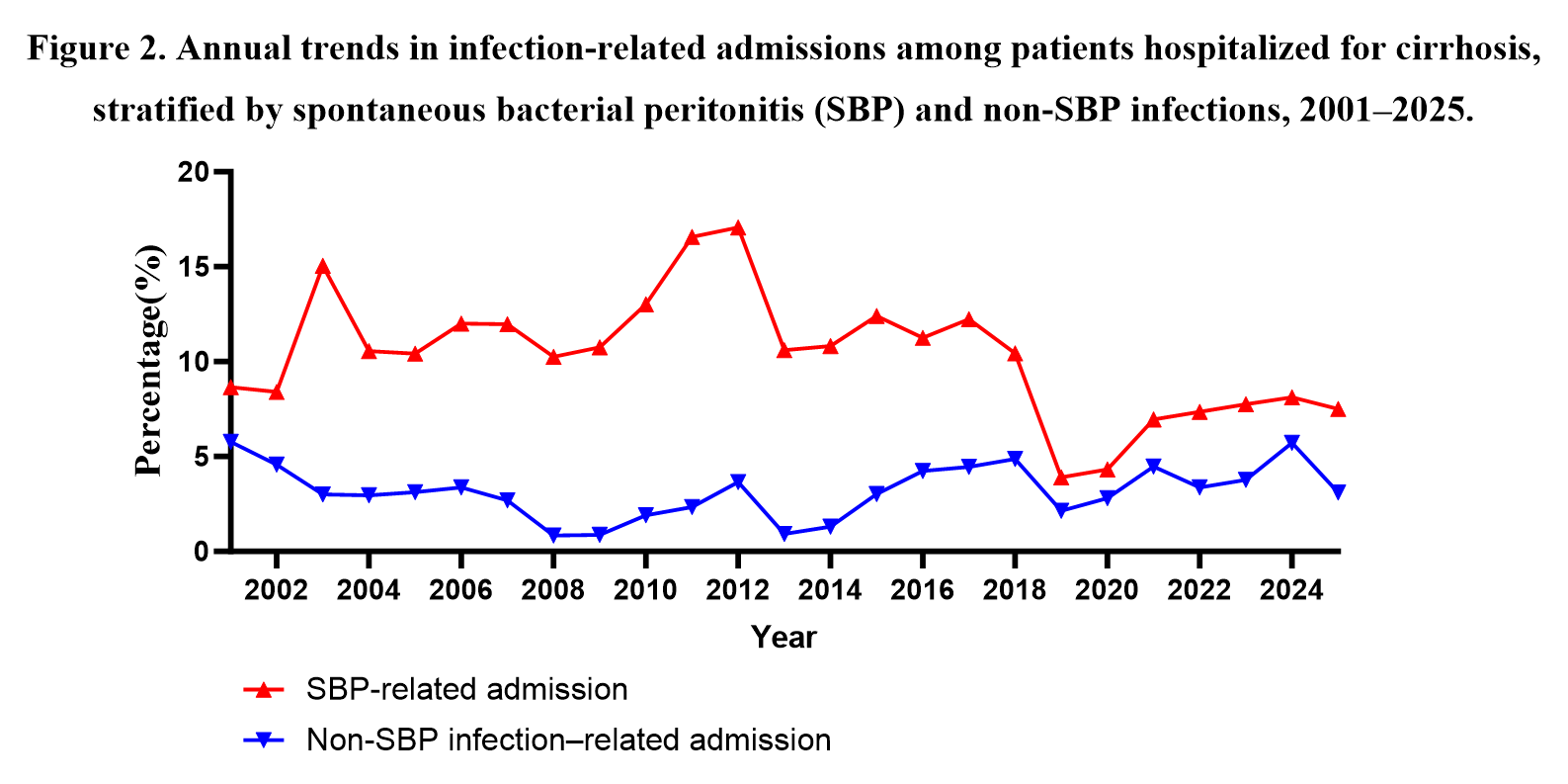

Supplement: Supplementary file 1 [file Table_1.DOCX]
